# (2,2′-Bi­pyridine-κ^2^*N*,*N*′)di­methyl­indium iodide

**DOI:** 10.1107/S2056989026010856

**Published:** 2026-10-30

**Authors:** Colby S. Bell, Taylor Harville, Toby J. Woods, Cong Liu, Karen L. Mulfort

**Affiliations:** a Division of Chemical Sciences and Engineering, Argonne National Laboratory, 9700 South Cass Ave, Lemont, Illinois 60439, USA; b University of Illinois, School of Chemical Sciences, Box 59-1, 505 South Mathews Avenue, Urbana, Illinois 61801, USA; Universidad de la Repüblica, Uruguay

**Keywords:** indium, organometallic, sil­yl, ligand-exchange, crystal structure

## Abstract

The reaction between a tetra­meric indium–sulfide cluster [InMe_4_(SSiMe_3_)]_4_ (**In_4_S_4_**, Me = meth­yl) and the copper dimer [CuI(bpy)]_2_ (bpy = 2,2′-bi­pyridine) yields the isolable title salt, [In(CH_3_)_2_(C_10_H_8_N_2_)]I or [InMe_2_(bpy)][I] (**1**); single-crystal X-ray diffraction, NMR, mass spectrometry, and elemental analysis confirm the structure and identity of compound **1**. Computational studies involving substitution at the 6,6′-bpy positions of **1** demonstrate steric control over THF solvent binding and suggest routes to tune reactivity pertinent for Lewis acid catalysis. Details inferred from the product isolated in this specific reaction, such as the propensity of indium to form the motif [InMe_2_(bpy)]^+^ can be used to inform future studies.

## Abstract 2

The crystal structure of (2,2′-bi­pyridine-κ^2^*N*,*N*′)di­methyl­indium iodide is reported. Structural comparisons are made with other di­methyl­indium species and computational studies of [InMe_2_(bpy)][I] derivatives examine the possibility of controlling metal accessibility through steric manipulation of the ligand sphere.

## 1. Chemical context

By replicating the efficiency endowed in natural discrete cluster systems such as ferredoxin and FeMoco, we hypothesized that new methods of energy transformation can be materialized to aid the rising needs of global demand (Tanifuji & Ohki, 2020 ▸; Ohki *et al.*, 2018 ▸; Le *et al.*, 2025 ▸). In light of this recognized potential, synthetic control over nuclearity, spatial arrangement, ligand environment, and hierarchical organization within cluster design is highly sought after (Ma *et al.*, 2025 ▸; Han *et al.*, 2022 ▸; Li *et al.*, 2026 ▸). Taking inspiration from the useful photocatalytic properties of In_2_S_3_ (Mishra *et al.*, 2023 ▸; 2024*a* ▸,*b*; 2024 ▸), our lab has sought to synthesize new discrete indium-sulfide clusters, leading to the isolation of [InMe_2_(SSiMe_3_)]_4_ (**In_4_S_4_**, Me = meth­yl) (Bell *et al.*, 2025 ▸). We found that **In_4_S_4_** is reactive with water and the neutral 4,4′-bi­pyridine ligand (Bell *et al.*, 2025 ▸); however, the optical absorption of the products were limited primarily to the UV region, restricting their viability toward initiating visible-light induced photochemical processes. While structurally informative, this previous work led us to further investigate the reactivity of **In_4_S_4_** to establish and predict the synthesis of new materials with promising optical activity, as a key challenge is the presence of multiple reactive groups and its ability to disproportionate into nonstoichiometric products (Bell *et al.*, 2025 ▸). To this end, we began to explore the behavior of **In_4_S_4_** with transition metal salts to examine trends in reactivity and simultaneously introduce structural and electronic complexity expected to enable lower energy optical transitions, provide sites to stabilize charge separation, and introduce (photo)catalytic active sites. Here we describe the reaction between **In_4_S_4_** and [CuI(2,2′-bi­pyridine)]_2_, ultimately leading to the crystallization of the title compound, [InMe_2_(2,2′-bi­pyridine)][I] (**1**).
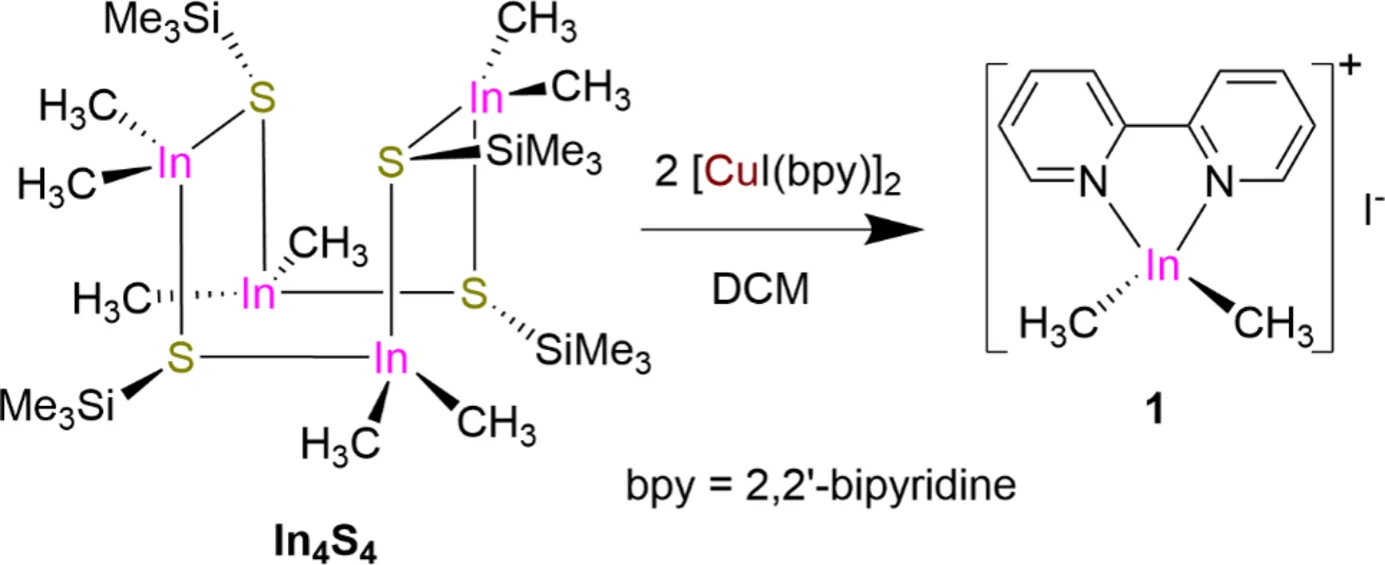


When two equivalents of [CuI(bpy)]_2_ (bpy = 2,2′-bi­pyridine) were introduced dropwise to **In_4_S_4_** in di­chloro­methane (DCM), the vibrant red color of the copper reagent immediately changed to pale yellow, indicating a fast reaction with **In_4_S_4_**. A pale-yellow crystalline powder was collected from the solution as the major product. Colorless block-shaped crystals suitable for scXRD analysis were obtained by vapor diffusion of pentane into a saturated tetra­hydro­furan (THF) solution of the pale-yellow crystalline powder, yielding [InMe_2_(bpy)][I] (**1**) whose mol­ecular structure is shown in Fig. 1 ▸. This species has been synthesized previously, either through addition of InMe_2_I to 2,2′-bi­pyridine or electrochemically using MeI, 2,2′-bi­pyridine and indium metal (Clark & Pickard, 1968 ▸; Habeeb *et al.*, 1980 ▸). However, a search of the Cambridge Structural Database (Version 2026.2, October 2026; Groom *et al.*, 2016 ▸) yielded no crystal structures matching that of complex **1**. NMR, mass spectrometry, and elemental analysis support the identification of **1**, and IR/UV-Vis data were collected to complete the characterization profile (see Figs. S1–S5). We have previously isolated the same cationic center [InMe_2_(bpy)]^+^ with a more complex anion, [(*μ*_3_-SSiMe_3_)(InMe_3_)_2_]^−^, from the reaction of InMe_3_(bpy) with S(SiMe_3_)_2_ (Bell *et al.*, 2025 ▸). This behavior suggests that the cation [InMe_2_(bpy)]^+^ is a highly stable motif and a favorable product for **In_4_S_4_** in the presence of 2,2′-bpy.

## 2. Structural commentary

Complex **1** crystallizes in the monoclinic *P*2_1_/*n* space group with the In1 atom adopting a distorted tetra­hedral geometry (Fig. 1 ▸). A salient feature of this structure is the unexpectedly large C11—In1—C12 angle of 157.55 (7)°, a 48° difference from the 109.5° expected from an idealized tetra­hedral geometry (Table 1 ▸). Comparison with other di­methyl­indium species helps to reveal a trend in the C—In—C angles, which seems to be responsive to the charge density of the counter-ion and steric ability to inter­act directly with the In center, in some cases generating pseudo five- or six-coordinate species. In **In_4_S_4_** the four C—In—C angles are 123–128°, aligned with the bond angles reported in similar structures including 129.5 (3)° observed in the dimer bis-pyrrolidinodi­methyl­indium, and 122–130° for the three angles present in the trimer [InMe_2_(*μ*-S-*t*-Bu)]_3_ (Bell *et al.*, 2025 ▸; Grafova *et al.*, 2004 ▸; Yearwood *et al.*, 2000 ▸). In structures where a coordinating anion is present, the C—In—C bond angle is influenced through short contact inter­actions, as seen in the significant increase to 144.84 (9)° observed in [InMe_2_(bpy)][(*μ*_3_-SSiMe_3_)(InMe_3_)_2_] (Bell *et al.*, 2025 ▸). Though more accurately considered an ion pair due to the elongated In—S bond, the C—In—C bond angle expansion by ∼20° signifies that an appreciable inter­action does occur between In and the anion, resulting in a pseudo five-coordinate species. Indeed, the even larger increase of the C—In—C bond angle to 157.55 (7)° in **1** can then be rationalized by the presence of two In⋯I short contacts (Fig. 2 ▸ top, discussed below), leading to In1 adopting pseudo-octa­hedral formation.

## 3. Supra­molecular features

Upon expansion of the asymmetric unit to fill the unit cell, we observe that each [InMe_2_(bpy)][I] unit is facing an adjacent mol­ecule. This orientation allows for two distinct In⋯I inter­actions at distances of 3.2963 (3) and 3.5214 (3) Å, resulting in a pseudo-octa­hedral geometry around the metal center (Fig. 2 ▸, top). The shortest In1⋯I1 distance found in **1**, however, is 0.528 Å longer than the average bond length of 2.768 Å in the distorted square-pyramidal structure InI_3_(TMEDA) (TMEDA = tetra­methyl­ethylenedi­amine) and 0.442 Å longer than the six- coordinate species *mer*-tri­iodo­tri­pyridine­indium, which has an average In—I distance of 2.8533 Å (Chitsaz *et al.*, 2002 ▸; Pardoe *et al.*, 2005 ▸). Comparison of these In—I bond lengths with those found in **1** suggest that the In—I inter­actions in **1** are composed of short contact inter­actions in the solid state as opposed to a formal bond. In addition to the In—I inter­actions, the centroid–centroid distances between the bpy ligands of neighboring mol­ecules are 3.6876 (14) and 3.9355 (11) Å, suggesting the presence of *π–π* stacking (Fig. 2 ▸, bottom).

## 4. Theoretical calculations and discussion

Cationic indium coordination complexes such as **1** have broad utility as Lewis acid catalysts, but a common limitation is that the large atomic radius of In allows for additional solvent coordination which can prevent substrate approach to the In active site (Goonesinghe *et al.*, 2023 ▸; Gabbaï *et al.*, 1996 ▸; Dagorne & Atwood, 2008 ▸). The prominent use of bi­pyridine ligands in coordination chemistry has resulted in many derivations that can be utilized to control electronic and/or steric inter­actions between the ligand and metal. Taking this into consideration we were motivated to explore how the bpy ligand may be derivatized to influence additional solvent coordination and the overall coordination environment of the In center. We hypothesized that since 2,2′-bi­pyridine is not particularly bulky compared to THF, it may be possible to control the In coordination environment through derivatization of the 6,6′-bpy positions. To capture this behavior, we calculated the thermodynamic binding energy of THF to the In center of **1** with increasingly bulky substituents at the 6,6′-bpy position, from H to methyl to ethyl to *tert*-butyl.

Given that **1** is an ion pair, and in practice the anion may be exchanged to fit the needs of the researcher, we have elected to model only the cation fragment to examine its behavior in the absence of an outside influence. For example, the bulky BArF anion, [B(C_6_F_5_)_4_]^−^, has been shown to have little to no inter­action with cationic In complexes (Goonesinghe *et al.*, 2023 ▸), but the short contact position assumed by I^−^ here clearly indicates that the coordinating ability of the anion has an appreciable impact on mol­ecular structure, which may influence catalytic activity. Therefore, the analyses concluding from these calculations are most likely to hold true for systems with noninter­acting anions.

In every case the binding of THF to the In center was found to be thermodynamically favorable. As expected, exchanging the hydrogen atoms for bulky substituents reduces the Gibbs free energy for THF binding to the In center (Table 2 ▸). Simply increasing from 6,6′-H to –CH_3_ results in a fivefold decrease in the free energy, from −8.48 to −1.66 kcal mol^−1^. We observe a slight increase in driving force for THF–In binding with ethyl substituents, which can be explained by the greater flexibility of the longer alkyl chain to accommodate the incoming solvent mol­ecule. The introduction of extreme bulk adjacent to the In center in the case of [InMe_2_(6,6′-*tert-*butyl-2,2′-bpy)]^+^ results in the most unfavorable In–THF inter­actions, explained by the large crowding effect from the *tert*-butyl substituents.

In all cases, it appears that THF binding to the In center results in a five-coordinate species (Fig. 3 ▸). The geometry about the In center differs significantly from both trigonal-bipyramidal and square-pyramidal and appears to be biased toward one or the other according to the extent of steric bulk. The *τ_5_* parameter is an index value that qu­anti­fies the degree of trigonality in five-coordinate systems inter­mediate of the limiting trigonal bipyramidal and square pyramidal possibilities (Addison *et al.*, 1984 ▸), and is calculated using the formula τ_5_ = (β - α)/(60°) where *β* and *α* are the two largest bond angles in the complex and *β* > *α.* A *τ_5_* value of 1 represents perfect trigonal-bipyramidal geometry, and *τ_5_* = 0 indicates perfect square-pyramidal geometry (Addison *et al.*, 1984 ▸). The *τ_5_* values for [InMe_2_(6,6′-*R*-bpy)(THF)]^+^ are 0.17, 0.46, 0.38, and 0.15 for *R* = H, methyl, ethyl, and *tert*-butyl, respectively (Table 2 ▸). The *τ_5_* values indicate that the In center adopts a nearly square-pyramidal geometry at the extremes of our series, either very little or substantial steric bulk with *R* = H or *tert-*butyl substitution, respectively. Substitution with methyl or ethyl results in a more inter­mediate *τ_5_* value, demonstrating the strong influence 6,6′-bpy substitution has on the In coordination environment. The N—C—C—N torsion angle of the bpy ligands is also highly responsive to the 6,6′-bpy sterics in this system. The N—C—C—-N angle of 5.95° in the H derivative decreases to 2.15 and 2.19° for the methyl and ethyl derivatives, respectively, but *tert-*butyl substitution results in a large distortion of the bpy ligand, resulting in an N—C—C—N angle of 39.2°.

The calculated In—O bond distance for *R* = H is 2.447 Å, consistent with experimentally observed distances of other five-coordinate In-based THF adducts such as [In(CH_2_SiMe_3_)_2_HF)_3_][B(C_6_F_5_)_4_] [2.424 (8) and 2.454 (8) Å] and (*o*-C_6_H_4_[InBr(THF)_2_])_2_ (2.489 and 2.430 Å) (Goonesinghe *et al.*, 2023 ▸; Gabbaï *et al.*, 1996 ▸). When the steric profile is increased to methyl, ethyl, and *tert*-butyl, the In—O bond is elongated to 2.674, 2.621, and 2.540 Å, respectively. The 0.1–0.2 Å In—O bond elongation compared to the 6,6′-H analogue indicates that the strength of solvent inter­action with the In center is highly responsive to the 6,6′-bpy substitution.

For the non-H 6,6′-bpy substituents, the In—O bond length decreases concomitant with increasing steric bulk, counter to our expectations as well as the trend observed in the Gibbs free energy calculations (Table 2 ▸). The alkyl substituents are weak sigma donors and will impact the coordinating ability of the 6,6′-*R*-2,2′-bpy ligand to the In center, although they all have similar Hammett parameters [*σ_p_* = −0.17 (Me), −0.15 (Et), −0.20 (*tert*-but­yl)] and the electronic donor effects in the substituted series are likely less of a factor in the calculated In—O distances than the steric factors (Hansch *et al.*, 1991 ▸). There are negligible changes to the In—C bonds lengths between derivatives, however, we do observe an increase in the calculated In—N bond lengths for the ethyl and *tert*-butyl substituents as compared to the H and methyl variants (Table 2 ▸).

Considering the coordination of the atoms (O, N, C) to the In center holistically, we note that the summed difference in In—O, In—N, and In—C bonding as compared to the H analog is greatest for the 6,6′-methyl version [Δ(In—O) + Δ(In—N) + Δ(In—C) = 0.219 Å] and is slightly less for the ethyl and *tert-*butyl complexes (0.190, 0.195, respectively). Therefore, the methyl version yields the most expanded and accessible In coordination environment based on computational analysis. These results provide a handle on how to effectively engineer the ligand environments of In-based Lewis acid catalysts to prevent unproductive solvent coordination while simultaneously preserving Lewis acidity and controlling overall geometry in catalyst design.

## 5. Conclusion

Overall, the isolation of **1** from the reaction between **In_4_S_4_** and [CuI(2,2′-bi­pyridine)]_2_ was unexpected and did not lead to a multimetallic species as envisioned; however, we have made two important observations that will help predict **In_4_S_4_** reactivity in future experiments. First, the In center is capable of undergoing anion exchange and acting as a leaving group to form mononuclear indium complexes. Secondly, the presence of a chelating ligand such as bpy clearly supports the formation of mononuclear species. The propensity to form motifs such as [InMe_2_(bpy)]^+^ must be considered in future reactions employing **In_4_S_4_**, which may be detrimental to efforts in isolating indium cluster species, but may also be beneficial in isolating new Lewis acid indium catalysts.

## 6. Experimental

### 6.1. General procedures

All manipulations were carried out under an inert N_2_ atmosphere using a glovebox or Schlenk techniques. [InMe_2_(SSiMe_3_)]_4_ and [CuI(bpy)]_2_ were synthesized using published procedures (Bell *et al.*, 2025 ▸; Skelton *et al.*, 1991 ▸). KBr (PIKE Technologies) was used as received without further purification. Pentane (Sigma-Aldrich), di­chloro­methane (Sigma-Aldrich), aceto­nitrile (VWR), tetra­hydro­furan (Sigma-Aldrich), and toluene (Sigma-Aldrich) were dried using 3 or 4 Å sieves and stored under an inert N_2_ atmosphere.

### 6.2. Physical measurements

NMR spectra were collected on a Bruker 500 MHz spectrometer in CD_2_Cl_2_ solvent at 298 K. ^1^H NMR spectra were referenced to TMS using the residual proteo impurities of the solvent; ^13^C{^1^H} NMR spectra were referenced to TMS using the natural abundance ^13^C{^1^H} impurities of the solvent. All chemical shifts are reported using the standard notation in parts-per-million referenced; positive chemical shifts are to a higher frequency from the given reference. Infrared spectra were recorded as KBr pellets on a Bruker Vertex 70v FT-IR spectrometer. Solution UV-visible absorption measurements were made on an Agilent Cary 60 UV-vis spectrophotometer. Mass spectral measurements were collected in positive ion mode on a ThermoFisher LCQ Fleet ESI instrument using aceto­nitrile solvent. Elemental analysis data were collected at the University of Illinois Urbana-Champaign Center of Research and Educational Support service facility.

### 6.3. Computational details

Geometry optimizations were carried out using density functional theory (DFT) as implemented in Orca, employing the B3LYP hybrid functional and the def2-TZVP basis set with the -GD4 dispersion correction (Becke, 1993 ▸; Stephens *et al.*, 1994 ▸; Weigend & Ahlrichs, 2005 ▸; Caldeweyher *et al.*, 2017 ▸). Reaction energies were calculated from single-point energy calculations at 298.15 K (25 °C) to reflect experimental conditions. Dispersion inter­actions were included using the DFT-D3 method with Becke–Johnson damping (Grimme *et al.*, 2010 ▸). Starting geometries were prepared using *Chemcraft* (graphical software for visualization of quantum chemistry computations; version 1.8, build 682) and *Vesta* (Momma & Izumi, 2011 ▸).

**[InMe_2_(bpy)][I] (1).** A 14 mL DCM slurry of [CuI(bpy)]_2_ (97.3 mg, 0.1403 mmol) was added dropwise to a stirring 2 mL DCM solution of [InMe_2_(SSiMe_3_)]_4_ (70.0 mg, 0.7000 mmol). After several minutes a brown precipitate was formed and the mixture was allowed to stir for an additional 3 days then filtered through a fine fritted funnel. The filtrate was evaporated to yield a light-yellow crystalline powder in 0.0913 g yield (76% based on [CuI(bpy)]_2_). Analysis calculated for C_12_H_14_N_2_InI: C 33.68%, H 3.30%, N 6.55%. Found: C 33.79%, H 3.27%, N 6.36%. MS (ESI^+^, ACN) *m/z* = theoretical: 301.02 [*M*^+^], actual: 301.08 [M^+^]. ^1^H NMR (CD_2_Cl_2_, 500 MHz) δ/ppm: 9.40 ppm (*d*, aryl-H, 2H), 8.24 (*d*, aryl-H, 2H), 8.12 (*t*, aryl-H, 2H), 7.66 (*t*, aryl-H, 2H), 0.16 (*s*, In—CH_3_, 6 H). ^13^C{^1^H} NMR (CD_2_Cl_2_, 500 MHz) δ/ppm: 150.37 (aryl-C), 150.24 (aryl-C), 140.12 (aryl-C), 126.04 (aryl-C), 122.10 (aryl-C), 3.48 (In—CH_3_). IR (KBr) *ν*/cm^−1^: 3109 (*w*), 3056 (*w*), 3023 (*w*), 3003 (*w*), 2983 (*w*), 2973 (*w*), 2901 (*w*), 1600 (*m*). 1593 (*m*), 1574 (*m*). 1562 (*sh*), 1489 (*w*), 1475 (*m*), 1436 (*s*), 1431 (*sh*), 1319 (*w*), 1310 (*w*), 1245 (*m*), 1213 (*w*), 1174 (*m*), 1156 (*m*), 1151 (*sh*), 1059 (*w*), 1039 (*w*), 1013 (*s*), 1007 (*sh*), 897 (*w*), 761 (s), 734 (*s*), 726 (*sh*), 649 (*w*), 644 (*m*), 625 (*m*), 541 (*m*). UV-Vis (CH_2_Cl_2_) [λ_max_ [nm] (ɛ, M^−1^ cm^−1^)]: 232 (93,000), 235 (88,000), 296 (52,000), 305 (45,000).

## 7. Refinement details

Intensity data were collected on a Bruker D8 Venture kappa diffractometer equipped with a Photon II (**1**) CPAD detector. An Iµs microfocus source provided the Mo *K*α radiation (*λ* = 0.71073 Å) that was monochromated with multilayer mirrors. The sample was mounted on a 0.3 mm nylon loop with the minimal amount of Paratone-N oil. Data were collected as a series of *φ* and/or *ω* scans. Data was collected at 100 K using a cold stream of N_2_(g) (Oxford Cryosytems Cryostream 700. The collection, cell refinement, and integration of intensity data was carried out with the *APEX3* software (Bruker, 2014 ▸). The absorption corrections were performed by multi-scan methods using *SADABS* (Krause *et al.*, 2015 ▸). The structure was solved with dual space methods using *SHELXT* version 2018/2 (Sheldrick, 2015*b* ▸). A structural model consisting of the target mol­ecule in the asymmetric unit was developed. The structure was refined with the full-matrix least-squares routine of *SHELXL* version 2019/3 (Sheldrick, 2015*a* ▸). H atoms were placed with a riding model for all the organic functional groups. Methyl H-atom positions, *R*—CH_3_, were optimized by rotation about *R*—C bonds with idealized C—H, *R*—H and H—H distances. Remaining H atoms were included as riding idealized contributors. Methyl H atom *U*_iso_(H) were assigned as 1.5 times *U*_eq_ of the carrier atom; remaining H atom *U*_iso_(H) were assigned as 1.2 times carrier *U*_eq_. The 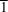 3 10 reflection was omitted from the final refinement due to being partially obscured by the beam stop support in some orientations. Crystal data, data collection and structure refinement details are summarized in Table 3 ▸.

## 8. Conflicts of inter­est

The authors declare no competing financial inter­est or conflict of inter­est.

## 9. Supporting information

Additional experimental characterization including NMR, IR, UV-Vis, and mass spectrometry analysis is available in the supporting information.

## Supplementary Material

Crystal structure: contains datablock(s) I, authors. DOI: 10.1107/S2056989026010856/ny2024sup1.cif

Structure factors: contains datablock(s) I. DOI: 10.1107/S2056989026010856/ny2024Isup2.hkl

Additional experimental characterization including NMR, IR, UV-Vis, and mass spectrometry analysis is available in the supporting information. DOI: 10.1107/S2056989026010856/ny2024sup3.pdf

CCDC reference: 2544428

Additional supporting information:  crystallographic information; 3D view; checkCIF report

## Figures and Tables

**Figure 1:**
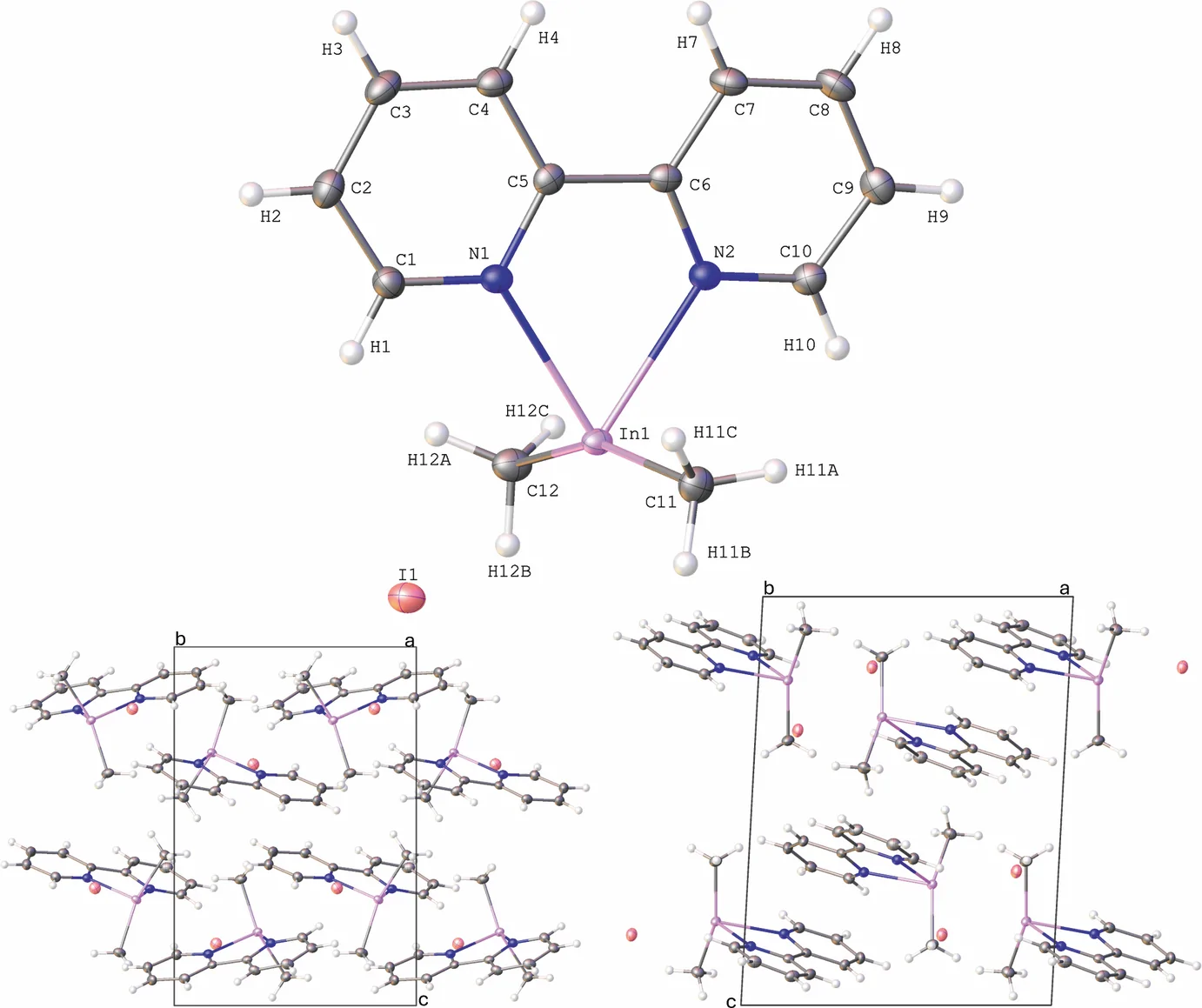
(Top) Crystal structure of [InMe2(bpy)][I] (**1**). Hydrogen atoms are omitted for clarity, displacement ellipsoids presented at 50% probability>. (Bottom) Depiction of the expanded unit cell of [InMe2(bpy)][I] viewed along the *a* (left) and *b* (right) axes. Unit cell depicted by black box.

**Figure 2:**
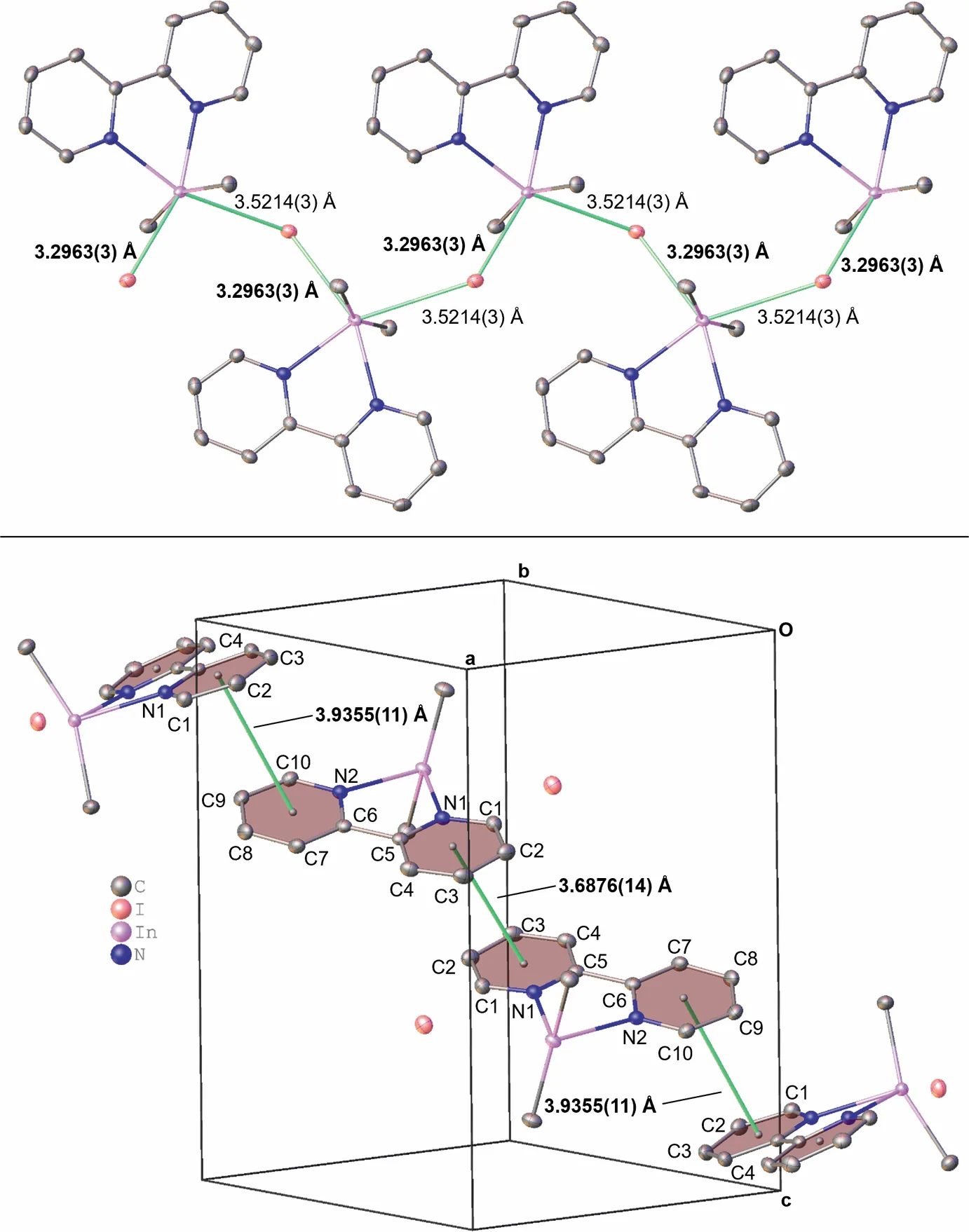
(Top) Short In1⋯I1 contacts (green). (Bottom) centroid–centroid distances between bpy ligands (green) within the unit cell. Hydrogen atoms omitted for clarity.

**Figure 3:**
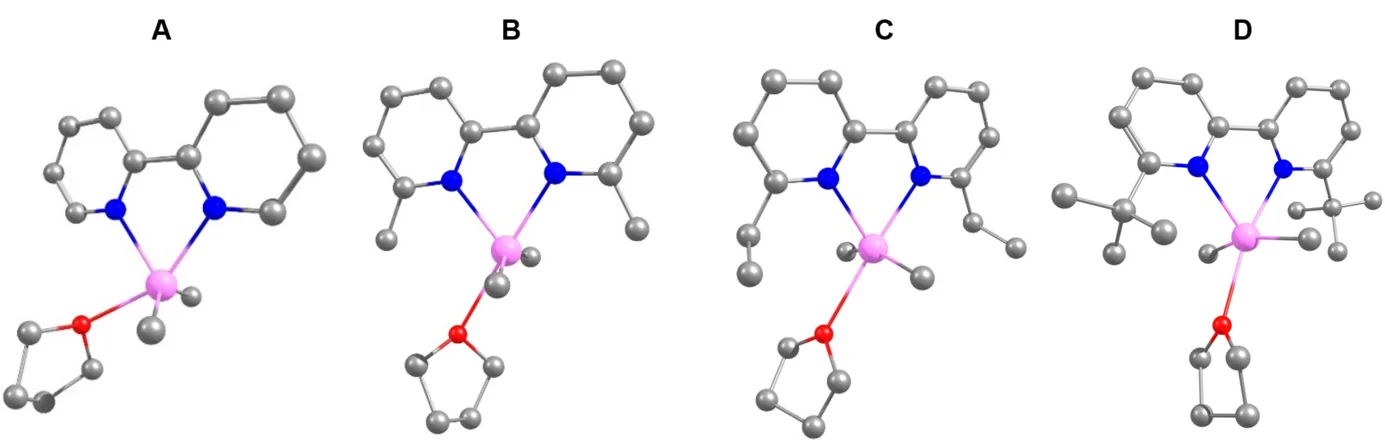
Gas phase DFT-optimized structures of [InMe_2_(6,6′-*R*-bpy)]^+^ [*R* = H (A), Me (B), Et (C), *tert*-butyl (D)] after THF coordination. Pink = indium, blue = nitro­gen, red = oxygen, gray = carbon. Hydrogen atoms omitted for clarity.

**Table 1:** Selected bond lengths (Å) and angles (°) of [InMe_2_(bpy)][I] (**1**)

In1—I1	3.2963 (3)	N1—In1—N2	68.43 (5)°
In1—I1^i^	3.5214 (3)	N1—In1—C11	105.72 (6)°
In1—C11	2.1303 (17)	N1—In1—C12	95.51 (6)°
In1—C12	2.1358 (17)	C11—In1—C12	157.55 (7)°
In1—N1	2.3827 (14)	N2—In1—C11	91.31 (6)°
In1—N2	2.4056 (14)	N2—In1—C12	103.45 (6)°
N1—C5	1.352 (2)		
N2—C6	1.351 (2)	π stacking (centroid distance)	3.6876 (14)
C5—C6	1.483 (2)	π stacking (centroid distance)	3.9355 (11)

Symmetry code: (i) 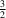 − *x*, −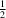 + *y*, 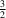 − *z*.

**Table 2:** Structural and thermodynamic parameters from gas phase DFT calculations of [InMe_2_(6,6′-*R*-bpy)(THF)]^+^ (*R* = H, methyl, ethyl, *tert*-but­yl)

*R* group	Δ*G* (kcal mol^−6^)	In—O (Å) / [+/- from H]	In—N_avg_ (Å) / [+/- from H]	In—C_avg_ (Å) / [+/- from H]	N—C—C–N (°)	C—In—C (°) / [τ]
H	−8.48	2.447 / [0]	2.341 / [0]	2.147 / [0]	5.95	141.7 / [0.17]
Meth­yl	−1.66	2.674 / [+0.227]	2.337 / [−0.004]	2.143 / [−0.004]	2.15	150.5 / [0.46]
Eth­yl	−2.15	2.621 / [+0.174]	2.361 / [+0.020]	2.143 / [−0.004]	2.19	153.5 / [0.38]
*tert*-but­yl	−1.46	2.540 / [+0.093]	2.445 / [+0.104]	2.145 / [−0.002]	39.2	158.2 / [0.15]

**Table 3:** Experimental details

Crystal data	
Chemical formula	[In(CH_3_)_2_(C_10_H_8_N_2_)]I
*M*_r_	427.97
Crystal system, space group	Monoclinic, *P*2_1_/*n*
Temperature (K)	100
*a*, *b*, *c* (Å)	10.5016 (3), 9.3267 (2), 13.8398 (3)
β (°)	93.073 (1)
*V* (Å^3^)	1353.59 (6)
*Z*	4
Radiation type	Mo *K*α
μ (mm^−1^)	4.00
Crystal size (mm)	0.18 × 0.14 × 0.11

Data collection	
Diffractometer	Bruker D8 Venture/Photon III
Absorption correction	Multi-scan (*SADABS*; Krause *et al.*, 2015 ▸)
*T*_min_, *T*_max_	0.592, 0.746
No. of measured, independent and observed [*I* > 2σ(*I*)] reflections	35437, 4115, 3855
*R*_int_	0.053
(sin θ/λ)_max_ (Å^−1^)	0.714

Refinement	
*R*[*F*^2^ > 2σ(*F*^2^)], *wR*(*F*^2^), *S*	0.019, 0.040, 1.07
No. of reflections	4115
No. of parameters	147
H-atom treatment	H-atom parameters constrained
Δρ_max_, Δρ_min_ (e Å^−3^)	0.78, −0.63

Computer programs: *APEX5*, *SAINT*, *XPREP* and *XCIF* (Bruker, 2024 ▸), *SHELXT2018/2* (Sheldrick, 2015*a* ▸), / *SHELXL20193* (Sheldrick, 2015*b* ▸), *SHELXTL* (Sheldrick, 2008 ▸) and *CrystalMaker* v9.2.8 (*CrystalMaker*, 2016 ▸).

## supplementary crystallographic information

### (2,2'-Bipyridine-κ2N,N')dimethylindium iodide Crystal data

**Table:**

[In(CH_3_)_2_(C_10_H_8_N_2_)]I	*F*(000) = 808
*M**_r_* = 427.97	*D*_x_ = 2.100 Mg m^−^^3^
Monoclinic, *P*2_1_/*n*	Mo *K*α radiation, λ = 0.71073 Å
*a* = 10.5016 (3) Å	Cell parameters from 18584 reflections
*b* = 9.3267 (2) Å	θ = 2.5–30.5°
*c* = 13.8398 (3) Å	µ = 4.00 mm^−^^1^
β = 93.073 (1)°	*T* = 100 K
*V* = 1353.59 (6) Å^3^	Block, colourless
*Z* = 4	0.18 × 0.14 × 0.11 mm

### (2,2'-Bipyridine-κ2N,N')dimethylindium iodide Data collection

**Table:**

Bruker D8 Venture/Photon III diffractometer	4115 independent reflections
Radiation source: microfocus sealed tube	3855 reflections with *I* > 2σ(*I*)
Multilayer mirrors monochromator	*R*_int_ = 0.053
profile data from φ and ω scans	θ_max_ = 30.5°, θ_min_ = 2.4°
Absorption correction: multi-scan (SADABS; Krause *et al.*, 2015)	*h* = −14→14
*T*_min_ = 0.592, *T*_max_ = 0.746	*k* = −12→13
35437 measured reflections	*l* = −19→15

### (2,2'-Bipyridine-κ2N,N')dimethylindium iodide Refinement

**Table:**

Refinement on *F*^2^	Primary atom site location: dual space
Least-squares matrix: full	Hydrogen site location: inferred from neighbouring sites
*R*[*F*^2^ > 2σ(*F*^2^)] = 0.019	H-atom parameters constrained
*wR*(*F*^2^) = 0.040	*w* = 1/[σ^2^(*F*_o_^2^) + (0.008*P*)^2^ + 1.4625*P*] where *P* = (*F*_o_^2^ + 2*F*_c_^2^)/3
*S* = 1.07	(Δ/σ)_max_ = 0.004
4115 reflections	Δρ_max_ = 0.78 e Å^−^^3^
147 parameters	Δρ_min_ = −0.63 e Å^−^^3^
0 restraints	

### (2,2'-Bipyridine-κ2N,N')dimethylindium iodide Special details

**Table:**

Experimental. One distinct cell was identified using APEX5 (Bruker, 2024). Eight frame series were integrated and filtered for statistical outliers using SAINT v8.40B (Bruker, 2024) then corrected for absorption by multi-scan methods using SADABS v2016/2 (Krause *et al.*, 2015). No decay correction was applied.
Geometry. All esds (except the esd in the dihedral angle between two l.s. planes) are estimated using the full covariance matrix. The cell esds are taken into account individually in the estimation of esds in distances, angles and torsion angles; correlations between esds in cell parameters are only used when they are defined by crystal symmetry. An approximate (isotropic) treatment of cell esds is used for estimating esds involving l.s. planes.
Refinement. Structure was phased by dual space methods (Sheldrick, 2015b). Systematic conditions suggested the unambiguous space group P2(1)/n. The space group choice was confirmed by successful convergence of the full-matrix least-squares refinement on F^2^. The final difference Fourier map had no significant features. A final analysis of variance between observed and calculated structure factors showed little dependence on amplitude or resolution.

### (2,2'-Bipyridine-κ2N,N')dimethylindium iodide Fractional atomic coordinates and isotropic or equivalent isotropic displacement parameters (Å^2^)

**Table:**

	*x*	*y*	*z*	*U*_iso_*/*U*_eq_	
I1	0.86439 (2)	0.32722 (2)	0.67250 (2)	0.01992 (4)	
In1	0.59529 (2)	0.15624 (2)	0.70485 (2)	0.01343 (4)	
N1	0.47830 (13)	0.35894 (15)	0.64422 (10)	0.0142 (3)	
N2	0.37301 (13)	0.10075 (15)	0.67465 (10)	0.0142 (3)	
C1	0.53047 (16)	0.48962 (18)	0.64153 (12)	0.0169 (3)	
H1	0.616704	0.501000	0.664620	0.020*	
C2	0.46444 (18)	0.60932 (19)	0.60656 (13)	0.0199 (3)	
H2	0.503852	0.701020	0.607008	0.024*	
C3	0.33979 (18)	0.59176 (19)	0.57105 (13)	0.0197 (3)	
H3	0.293262	0.670763	0.543958	0.024*	
C4	0.28344 (17)	0.45761 (19)	0.57540 (12)	0.0177 (3)	
H4	0.197427	0.444045	0.552402	0.021*	
C5	0.35463 (16)	0.34267 (17)	0.61399 (12)	0.0140 (3)	
C6	0.29787 (15)	0.19908 (18)	0.62811 (12)	0.0137 (3)	
C7	0.17233 (16)	0.16769 (19)	0.59826 (13)	0.0170 (3)	
H7	0.120237	0.238367	0.566388	0.020*	
C8	0.12402 (16)	0.0324 (2)	0.61547 (13)	0.0193 (3)	
H8	0.038195	0.009610	0.596219	0.023*	
C9	0.20219 (17)	−0.06927 (19)	0.66102 (12)	0.0182 (3)	
H9	0.171859	−0.163405	0.672396	0.022*	
C10	0.32591 (16)	−0.03025 (18)	0.68964 (12)	0.0164 (3)	
H10	0.379666	−0.099594	0.721303	0.020*	
C11	0.64444 (17)	0.03756 (19)	0.58071 (13)	0.0194 (3)	
H11A	0.595829	−0.052156	0.577390	0.029*	
H11B	0.735880	0.015925	0.585326	0.029*	
H11C	0.624213	0.094332	0.522303	0.029*	
C12	0.60616 (17)	0.2256 (2)	0.85214 (12)	0.0196 (3)	
H12A	0.603616	0.330552	0.854434	0.029*	
H12B	0.686027	0.191515	0.884100	0.029*	
H12C	0.533809	0.186479	0.885455	0.029*	

### (2,2'-Bipyridine-κ2N,N')dimethylindium iodide Atomic displacement parameters (Å^2^)

**Table:**

	*U*^11^	*U*^22^	*U*^33^	*U*^12^	*U*^13^	*U*^23^
I1	0.01493 (6)	0.02177 (6)	0.02290 (7)	0.00206 (4)	−0.00045 (4)	−0.00057 (4)
In1	0.01263 (6)	0.01514 (6)	0.01233 (6)	0.00232 (4)	−0.00113 (4)	−0.00198 (4)
N1	0.0134 (6)	0.0166 (6)	0.0125 (6)	0.0014 (5)	0.0003 (5)	−0.0004 (5)
N2	0.0136 (6)	0.0161 (6)	0.0127 (6)	0.0009 (5)	0.0002 (5)	0.0002 (5)
C1	0.0160 (7)	0.0175 (7)	0.0171 (8)	−0.0005 (6)	−0.0001 (6)	−0.0014 (6)
C2	0.0261 (9)	0.0147 (7)	0.0188 (8)	0.0008 (6)	0.0015 (7)	0.0001 (6)
C3	0.0259 (9)	0.0161 (8)	0.0168 (8)	0.0062 (6)	−0.0019 (7)	0.0003 (6)
C4	0.0177 (8)	0.0198 (8)	0.0154 (8)	0.0044 (6)	−0.0017 (6)	−0.0006 (6)
C5	0.0166 (7)	0.0149 (7)	0.0105 (7)	0.0017 (6)	0.0016 (6)	−0.0015 (6)
C6	0.0130 (7)	0.0176 (7)	0.0106 (7)	0.0020 (6)	0.0018 (5)	−0.0008 (6)
C7	0.0132 (7)	0.0227 (8)	0.0149 (8)	0.0011 (6)	−0.0005 (6)	0.0031 (6)
C8	0.0122 (7)	0.0276 (9)	0.0180 (8)	−0.0035 (6)	0.0007 (6)	0.0013 (7)
C9	0.0183 (8)	0.0211 (8)	0.0155 (8)	−0.0036 (6)	0.0042 (6)	0.0010 (6)
C10	0.0169 (7)	0.0175 (7)	0.0149 (7)	0.0010 (6)	0.0022 (6)	0.0019 (6)
C11	0.0211 (8)	0.0202 (8)	0.0172 (8)	0.0011 (6)	0.0027 (6)	−0.0031 (6)
C12	0.0194 (8)	0.0250 (9)	0.0141 (8)	0.0037 (7)	−0.0010 (6)	−0.0032 (7)

### (2,2'-Bipyridine-κ2N,N')dimethylindium iodide Geometric parameters (Å, º)

**Table:**

In1—C11	2.1303 (17)	C5—C6	1.483 (2)
In1—C12	2.1358 (17)	C6—C7	1.391 (2)
In1—N1	2.3827 (14)	C7—C8	1.386 (2)
In1—N2	2.4056 (14)	C7—H7	0.9500
N1—C1	1.338 (2)	C8—C9	1.383 (3)
N1—C5	1.352 (2)	C8—H8	0.9500
N2—C10	1.338 (2)	C9—C10	1.387 (2)
N2—C6	1.351 (2)	C9—H9	0.9500
C1—C2	1.388 (2)	C10—H10	0.9500
C1—H1	0.9500	C11—H11A	0.9800
C2—C3	1.383 (3)	C11—H11B	0.9800
C2—H2	0.9500	C11—H11C	0.9800
C3—C4	1.387 (3)	C12—H12A	0.9800
C3—H3	0.9500	C12—H12B	0.9800
C4—C5	1.397 (2)	C12—H12C	0.9800
C4—H4	0.9500		
			
C11—In1—C12	157.55 (7)	N2—C6—C7	121.25 (15)
C11—In1—N1	105.72 (6)	N2—C6—C5	116.57 (14)
C12—In1—N1	95.51 (6)	C7—C6—C5	122.14 (15)
C11—In1—N2	91.31 (6)	C8—C7—C6	119.37 (16)
C12—In1—N2	103.45 (6)	C8—C7—H7	120.3
N1—In1—N2	68.43 (5)	C6—C7—H7	120.3
C1—N1—C5	118.81 (14)	C9—C8—C7	119.24 (16)
C1—N1—In1	121.91 (11)	C9—C8—H8	120.4
C5—N1—In1	119.25 (11)	C7—C8—H8	120.4
C10—N2—C6	118.84 (14)	C8—C9—C10	118.32 (16)
C10—N2—In1	122.19 (11)	C8—C9—H9	120.8
C6—N2—In1	118.26 (11)	C10—C9—H9	120.8
N1—C1—C2	123.03 (16)	N2—C10—C9	122.94 (16)
N1—C1—H1	118.5	N2—C10—H10	118.5
C2—C1—H1	118.5	C9—C10—H10	118.5
C3—C2—C1	118.32 (16)	In1—C11—H11A	109.5
C3—C2—H2	120.8	In1—C11—H11B	109.5
C1—C2—H2	120.8	H11A—C11—H11B	109.5
C2—C3—C4	119.29 (16)	In1—C11—H11C	109.5
C2—C3—H3	120.4	H11A—C11—H11C	109.5
C4—C3—H3	120.4	H11B—C11—H11C	109.5
C3—C4—C5	119.23 (16)	In1—C12—H12A	109.5
C3—C4—H4	120.4	In1—C12—H12B	109.5
C5—C4—H4	120.4	H12A—C12—H12B	109.5
N1—C5—C4	121.19 (15)	In1—C12—H12C	109.5
N1—C5—C6	116.55 (14)	H12A—C12—H12C	109.5
C4—C5—C6	122.20 (15)	H12B—C12—H12C	109.5
			
C5—N1—C1—C2	−1.8 (3)	C10—N2—C6—C5	179.64 (14)
In1—N1—C1—C2	179.98 (13)	In1—N2—C6—C5	−9.80 (18)
N1—C1—C2—C3	−1.4 (3)	N1—C5—C6—N2	3.6 (2)
C1—C2—C3—C4	2.8 (3)	C4—C5—C6—N2	−173.73 (15)
C2—C3—C4—C5	−1.1 (3)	N1—C5—C6—C7	−178.63 (15)
C1—N1—C5—C4	3.6 (2)	C4—C5—C6—C7	4.1 (2)
In1—N1—C5—C4	−178.17 (12)	N2—C6—C7—C8	−0.9 (3)
C1—N1—C5—C6	−173.77 (15)	C5—C6—C7—C8	−178.57 (16)
In1—N1—C5—C6	4.50 (19)	C6—C7—C8—C9	−0.7 (3)
C3—C4—C5—N1	−2.1 (3)	C7—C8—C9—C10	1.3 (3)
C3—C4—C5—C6	175.06 (16)	C6—N2—C10—C9	−1.2 (2)
C10—N2—C6—C7	1.8 (2)	In1—N2—C10—C9	−171.36 (13)
In1—N2—C6—C7	172.38 (12)	C8—C9—C10—N2	−0.4 (3)
