# Supplementary material for: (2,2′-Bi­pyridine-κ2N,N′)di­methyl­indium iodide
Source: Acta Crystallogr E Crystallogr Commun. 2026 Oct 30;82(Pt 11):1346–51. doi: 10.1107/S2056989026010856 (PMC13741340; doi:10.1107/S2056989026010856)
Supplement: Supplementary file 1 [file e-82-01346-sup3.pdf]

Supporting information

Supporting information that may be of use or interest to some readers will be made available from the IUCr archives and appropriate databases.

S1. Nuclear Magnetic Resonance Spectroscopy

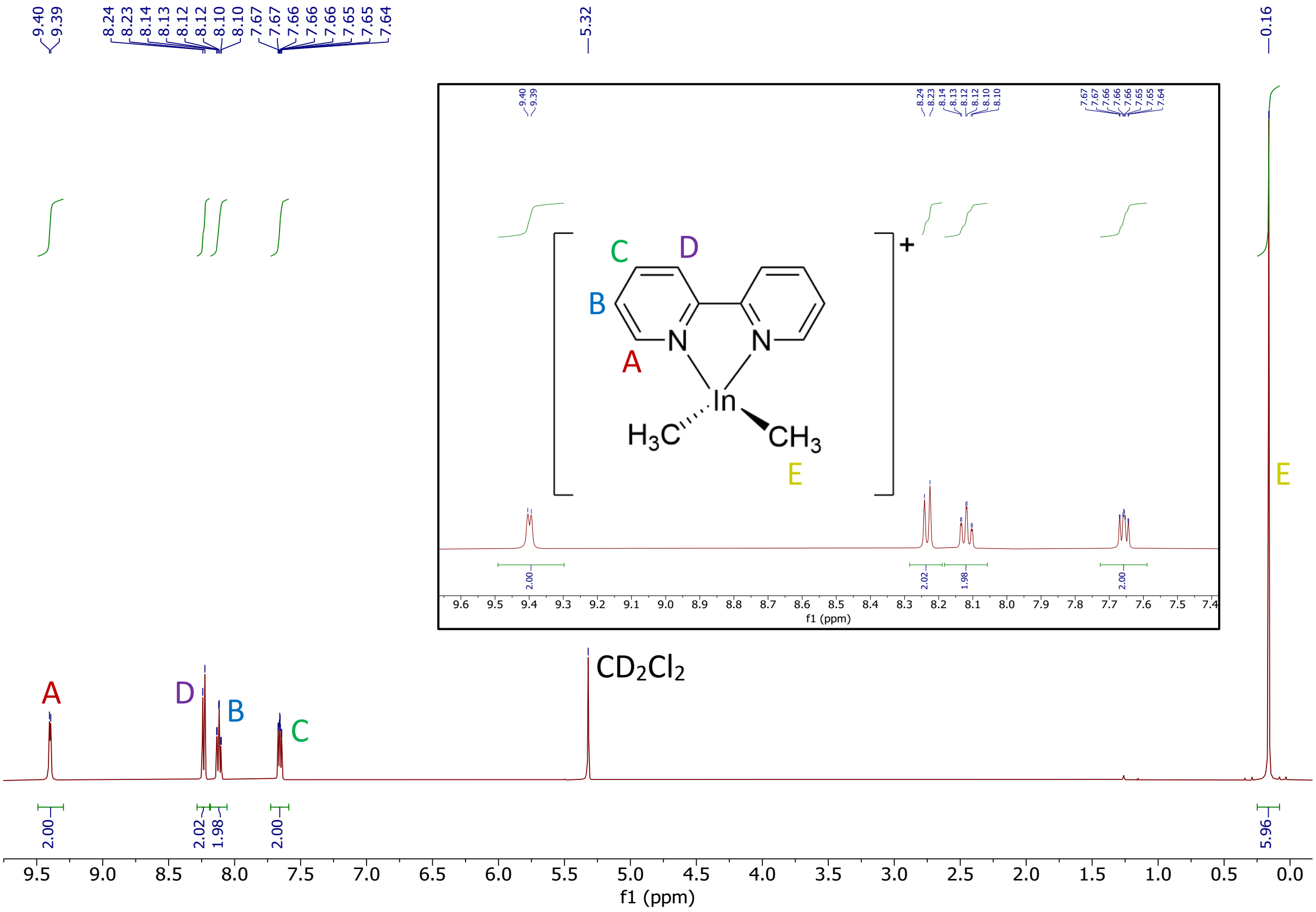

Figure S1 ^{1}H NMR of [InMe$_2$(bpy)][I] (**1**) in CD_2Cl_2 at 298 K.

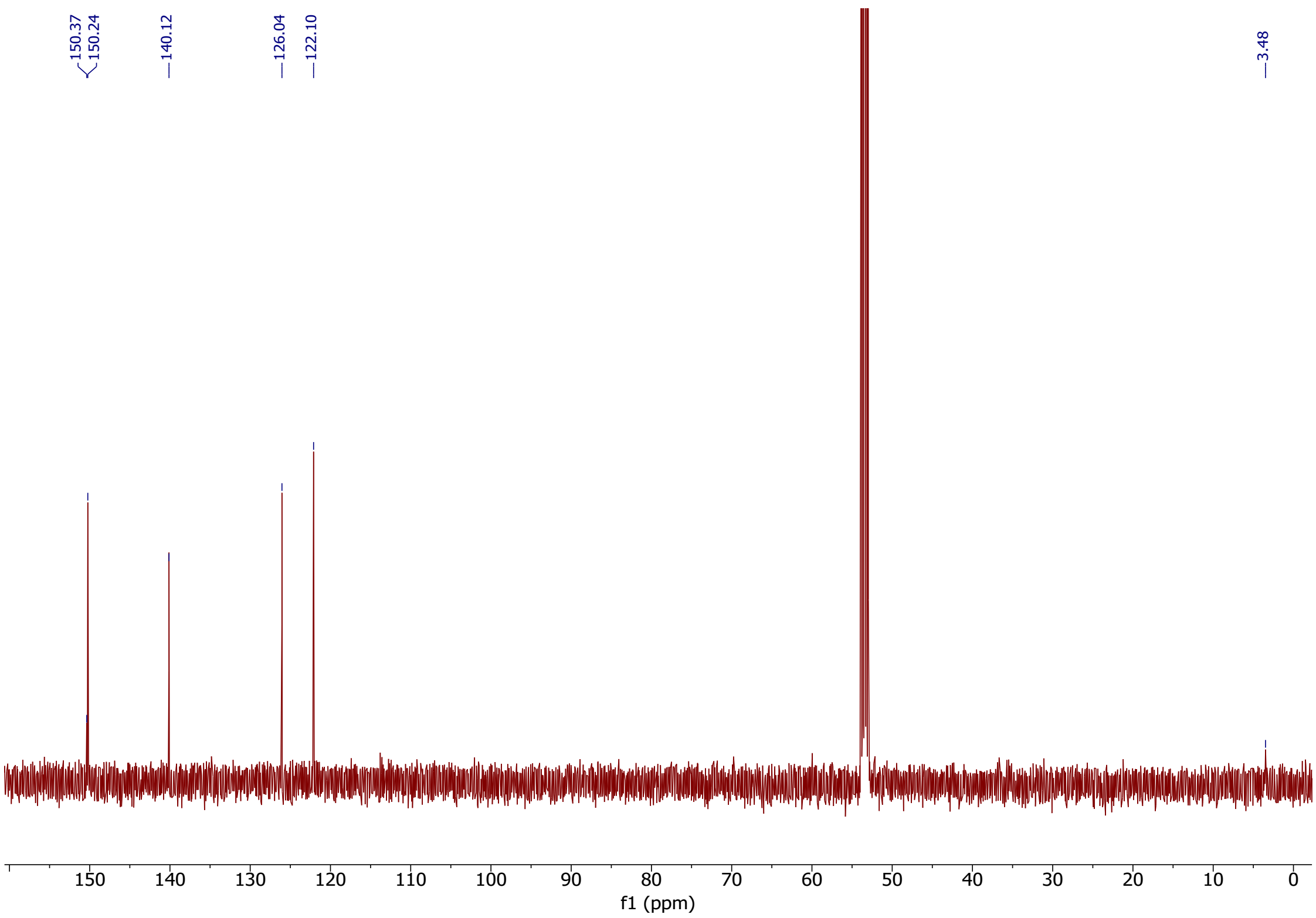

Figure S2 $^{13}C\{^1H\}$ NMR of $[InMe_2(bpy)][I]$ (**1**) in CD_2Cl_2 at 298 K.

S2. Infrared Vibrational Spectroscopy

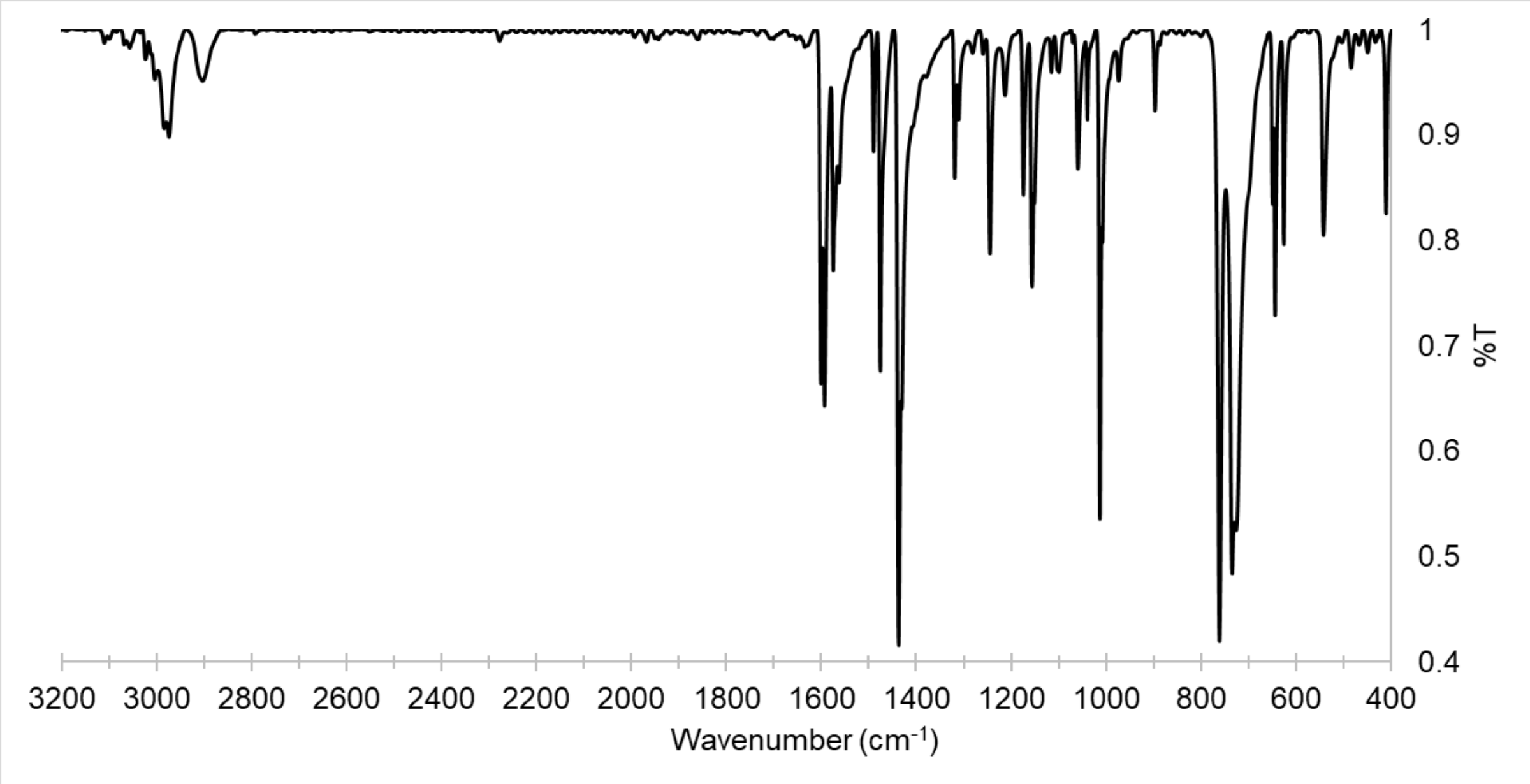

Figure S3 Infrared spectrum of $[InMe_2(bpy)][I]$ (**1**) in a KBr pellet at 298 K.

S3. UV-Visible Absorption Spectroscopy

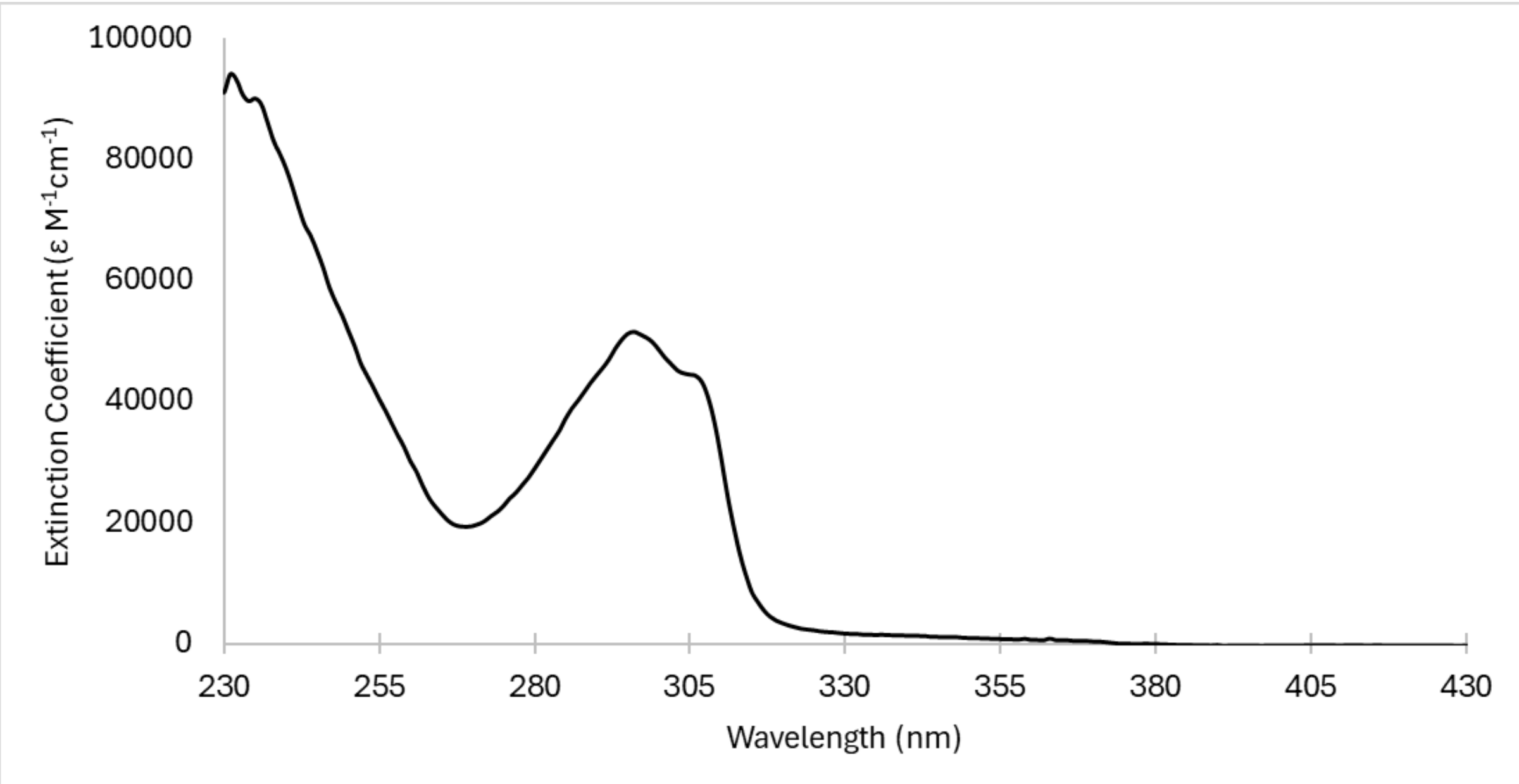

Figure S4 UV-Vis spectrum of $[InMe_2(bpy)][I]$ (**1**) in CH_2Cl_2 at 298 K.

S4. Mass Spectrometry

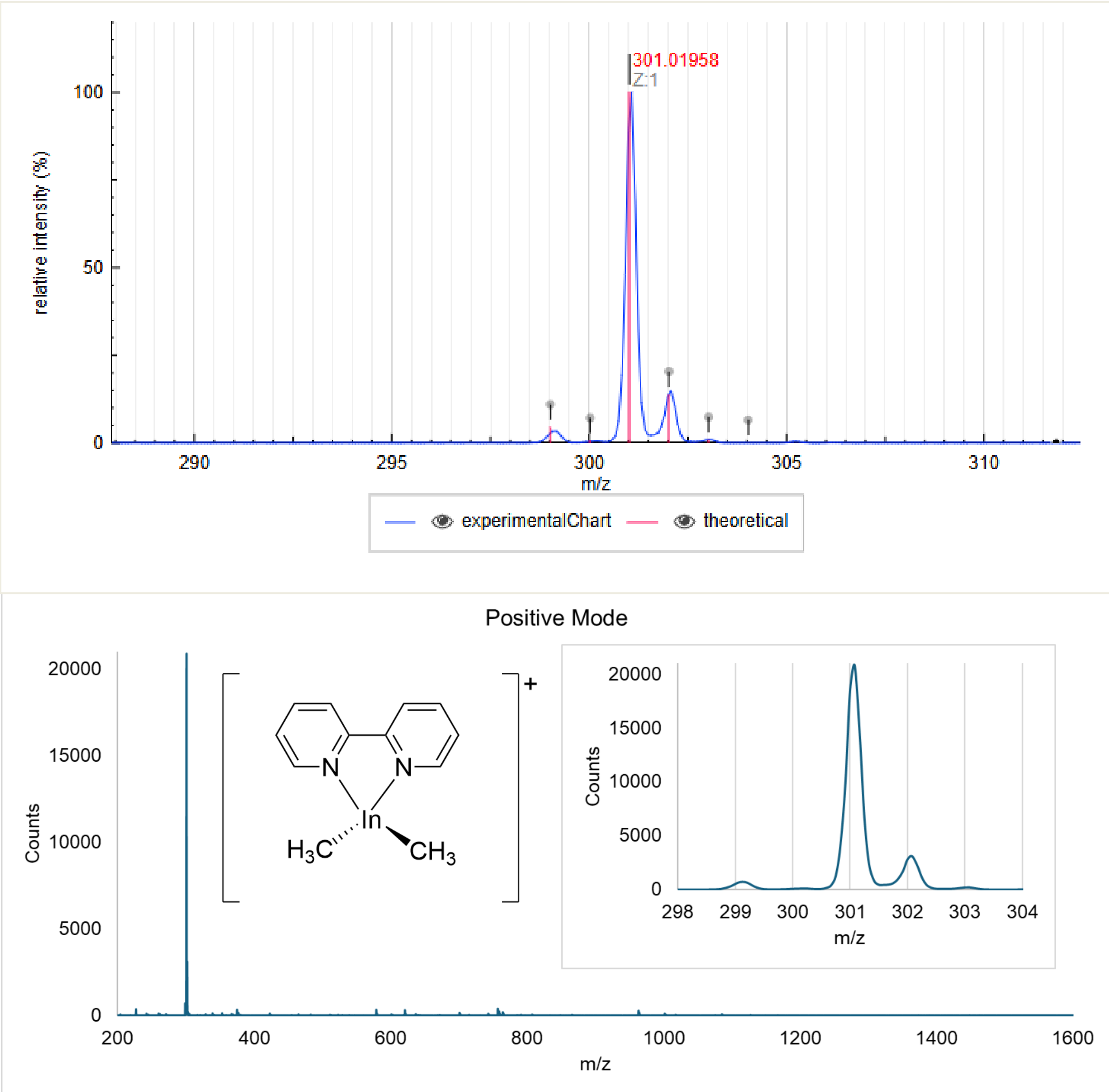

Figure S5 Mass spectrum (ESI, positive mode) simulation (top) and raw data (bottom) of $[InMe_2(bpy)][I]$ (**1**) in CH_3CN at 298 K.

S5. Computational Calculations – Atomic coordinates of modeled [In(6,6'-R-2,2'-bpy)$(CH_3)_2$]$^+$ complexes

XYZ coordinates for [$InMe_2$(2,2′-bipyridine)(THF)]$^+$

C	0.155355000	3.674116000	-0.284023000
C	2.627950000	0.226206000	0.181794000
C	3.997855000	0.238668000	0.492246000
C	4.692385000	-0.977100000	0.566002000
C	4.009770000	-2.180475000	0.331567000
C	2.641518000	-2.121001000	0.036010000
N	1.983312000	-0.946152000	-0.037197000
H	4.518699000	1.167527000	0.682568000
H	5.749026000	-0.983021000	0.805226000
H	4.516177000	-3.135296000	0.380095000
H	2.062326000	-3.020761000	-0.143109000
C	1.801364000	1.460710000	0.056500000
C	2.356029000	2.749437000	0.105540000
C	1.525918000	3.866166000	-0.059581000
C	-0.339592000	2.364655000	-0.318655000
N	0.464242000	1.291096000	-0.139641000
H	3.418590000	2.886441000	0.253875000
H	1.945396000	4.864488000	-0.025652000
H	-0.514891000	4.510153000	-0.433333000
H	-1.384794000	2.150673000	-0.503097000
In	-0.366627000	-0.864473000	-0.305293000
C	-0.714276000	-1.834176000	1.582377000
H	-0.385152000	-1.195723000	2.407569000
H	-1.777380000	-2.057209000	1.711656000
H	-0.157925000	-2.773899000	1.640092000
C	-0.663427000	-1.128762000	-2.411136000
H	-1.042287000	-0.192349000	-2.832015000
H	0.277060000	-1.375424000	-2.908910000
H	-1.385176000	-1.920976000	-2.622950000
H	-3.146010000	1.808816000	1.202733000
O	-2.529907000	0.249793000	-0.049717000
C	-3.612790000	-0.450408000	-0.757823000
C	-4.871228000	-0.120567000	0.044878000
C	-4.322193000	-0.012036000	1.477662000
C	-3.001462000	0.725007000	1.257180000
H	-3.627925000	-0.104859000	-1.791413000
H	-3.395683000	-1.525098000	-0.741620000
H	-5.639613000	-0.886052000	-0.059280000
H	-5.295888000	0.833961000	-0.276496000
H	-4.138111000	-1.007248000	1.892816000
H	-4.990493000	0.520952000	2.153326000
H	-2.233913000	0.498499000	1.999641000

XYZ coordinates for [$InMe_2$(6,6'-dimethyl-2,2'-bipyridine)(THF)]$^+$

C	1.162602000	3.865621000	-0.094550000
C	2.785731000	-0.074315000	0.088700000
C	4.154012000	-0.371135000	0.181146000
C	4.554644000	-1.714011000	0.223729000
C	3.590076000	-2.727191000	0.167728000
C	2.230635000	-2.376517000	0.075741000
N	1.866028000	-1.071143000	0.045349000
H	4.897663000	0.412521000	0.222200000
H	5.606507000	-1.963559000	0.297101000
H	3.872302000	-3.772061000	0.193055000
C	2.260595000	1.321122000	0.027822000
C	3.109614000	2.437053000	0.013737000
C	2.553197000	3.721327000	-0.050231000
C	0.349819000	2.718385000	-0.075850000
N	0.907547000	1.480511000	-0.020237000
H	4.183426000	2.317875000	0.046609000
H	3.196683000	4.592902000	-0.065141000
H	0.700749000	4.843438000	-0.141777000
In	-0.398456000	-0.434599000	-0.078193000
C	-0.937597000	-0.839158000	1.956245000
H	-0.170262000	-1.436115000	2.455775000
H	-1.043103000	0.102854000	2.503469000
H	-1.892214000	-1.371102000	1.996616000
C	-0.645964000	-0.783516000	-2.177041000
H	-1.240085000	0.027473000	-2.607633000
H	0.331792000	-0.808367000	-2.664566000
H	-1.162742000	-1.726509000	-2.374411000
H	-4.196556000	1.748030000	0.487041000
O	-2.992836000	0.196482000	-0.231240000
C	-3.711510000	-0.892668000	-0.896773000
C	-5.155177000	-0.773137000	-0.404799000
C	-4.962794000	-0.230406000	1.020253000
C	-3.826047000	0.774381000	0.824087000
H	-3.597093000	-0.778735000	-1.974877000
H	-3.262690000	-1.846950000	-0.589932000
H	-5.684860000	-1.725186000	-0.432353000
H	-5.710342000	-0.055919000	-1.014809000
H	-4.647378000	-1.032929000	1.694140000
H	-5.862005000	0.227339000	1.431805000
H	-3.211608000	0.911782000	1.716223000
C	-1.148588000	2.809129000	-0.095006000
H	-1.580144000	2.209966000	-0.902060000
H	-1.562136000	2.422890000	0.842201000
H	-1.482534000	3.839473000	-0.212764000
C	1.139052000	-3.408794000	-0.004611000

H	0.575989000	-3.300183000	-0.937114000
H	1.544385000	-4.419459000	0.029239000
H	0.434491000	-3.298552000	0.825580000

XYZ coordinates for [InMe$_2$(6,6'-diethyl-2,2'-bipyridine)(THF)]$^+$

C	-0.213708000	4.029275000	0.057811000
C	-2.755552000	0.603991000	-0.024319000
C	-4.146497000	0.668032000	-0.189870000
C	-4.897579000	-0.515264000	-0.119876000
C	-4.246599000	-1.727675000	0.119768000
C	-2.845486000	-1.740754000	0.270429000
N	-2.138177000	-0.589149000	0.190136000
H	-4.648873000	1.607992000	-0.369930000
H	-5.973493000	-0.483200000	-0.244061000
H	-4.796696000	-2.658585000	0.190566000
C	-1.890731000	1.821269000	-0.039429000
C	-2.432642000	3.108079000	-0.171080000
C	-1.584318000	4.222830000	-0.130953000
C	0.287180000	2.720641000	0.181624000
N	-0.545127000	1.649709000	0.115770000
H	-3.496736000	3.251828000	-0.292668000
H	-1.992244000	5.221537000	-0.231510000
H	0.472236000	4.865791000	0.113660000
In	0.245245000	-0.549789000	0.151362000
C	0.333031000	-1.021841000	-1.939440000
H	-0.654703000	-0.870076000	-2.383202000
H	1.050395000	-0.372555000	-2.448534000
H	0.636186000	-2.059489000	-2.102873000
C	0.592726000	-0.957567000	2.223576000
H	1.171989000	-0.147481000	2.677277000
H	-0.360612000	-1.038662000	2.751658000
H	1.151061000	-1.888889000	2.347994000
H	3.644079000	-0.236294000	-1.885300000
O	2.861749000	-0.630042000	0.012737000
C	3.776074000	-0.548135000	1.153689000
C	5.170725000	-0.544851000	0.527596000
C	4.975366000	-1.481293000	-0.675179000
C	3.588952000	-1.073472000	-1.181756000
H	3.545939000	0.354961000	1.722884000
H	3.621669000	-1.419248000	1.797961000
H	5.941240000	-0.887192000	1.218071000
H	5.435657000	0.461640000	0.189648000
H	4.975015000	-2.524189000	-0.347611000
H	5.745340000	-1.365472000	-1.437696000
H	3.034633000	-1.889197000	-1.646185000
C	1.763568000	2.480646000	0.354293000

H	1.919298000	1.553487000	0.911908000
H	2.193395000	3.289261000	0.951458000
C	-2.115810000	-3.045667000	0.475769000
H	-1.120606000	-2.856768000	0.889808000
H	-2.649536000	-3.643496000	1.220436000
C	2.483123000	2.393470000	-1.002743000
H	3.538856000	2.150969000	-0.858680000
H	2.043242000	1.607888000	-1.623085000
H	2.417640000	3.337974000	-1.548705000
C	-2.010728000	-3.838901000	-0.840325000
H	-1.486677000	-3.263214000	-1.606909000
H	-1.469834000	-4.773940000	-0.685475000
H	-3.002310000	-4.086199000	-1.228414000

XYZ coordinates for [$InMe_2$(6,6’-*tert*-butyl-2,2’-bipyridine)(THF)]$^+$

C	-4.393068000	0.215399000	-0.873826000
C	-0.877265000	2.414680000	0.045275000
C	-1.060819000	3.764839000	0.382892000
C	0.057880000	4.554636000	0.656928000
C	1.328705000	3.986202000	0.534934000
C	1.475718000	2.626412000	0.200722000
N	0.364030000	1.848636000	0.011576000
H	-2.060682000	4.171445000	0.455267000
H	-0.055269000	5.593423000	0.943737000
H	2.205530000	4.594361000	0.699014000
C	-2.092804000	1.618049000	-0.314714000
C	-3.115148000	2.252809000	-1.039767000
C	-4.270533000	1.527214000	-1.344434000
C	-3.343178000	-0.379928000	-0.146450000
N	-2.192912000	0.321896000	0.075858000
H	-2.990876000	3.271084000	-1.384148000
H	-5.065490000	1.976912000	-1.927679000
H	-5.295099000	-0.343864000	-1.074012000
In	0.145942000	-0.538767000	0.041820000
C	0.247705000	-0.891195000	2.155605000
H	-0.350967000	-0.118950000	2.647105000
H	-0.174755000	-1.868756000	2.406380000
H	1.265534000	-0.846796000	2.544867000
C	-0.149433000	-0.973365000	-2.037975000
H	-0.073958000	-2.050869000	-2.208983000
H	-1.148695000	-0.641698000	-2.335426000
H	0.585727000	-0.463477000	-2.665707000
H	1.957356000	-3.230469000	1.418790000
O	2.276950000	-1.906992000	-0.155559000
C	2.988700000	-2.283167000	-1.376678000
C	3.448652000	-3.712138000	-1.101972000

C	3.857118000	-3.620968000	0.378396000
C	2.794906000	-2.685745000	0.976358000
H	2.302730000	-2.175792000	-2.215739000
H	3.842154000	-1.610664000	-1.518293000
H	4.267706000	-4.024224000	-1.749701000
H	2.618017000	-4.410070000	-1.235322000
H	4.852679000	-3.178237000	0.465398000
H	3.877469000	-4.590509000	0.875543000
H	3.202604000	-1.999784000	1.723100000
C	-3.495525000	-1.774928000	0.469459000
C	2.872429000	2.025484000	0.001020000
C	-4.862108000	-2.398415000	0.137583000
H	-5.691828000	-1.786615000	0.502567000
H	-4.989948000	-2.567093000	-0.936115000
H	-4.932775000	-3.369062000	0.634521000
C	-2.408963000	-2.747324000	-0.030476000
H	-2.326253000	-2.743943000	-1.120604000
H	-1.430225000	-2.527504000	0.404669000
H	-2.666959000	-3.760861000	0.289209000
C	-3.392271000	-1.629582000	2.002749000
H	-3.438544000	-2.618446000	2.468311000
H	-2.454064000	-1.152825000	2.289804000
H	-4.218591000	-1.029891000	2.395304000
C	2.926775000	1.297068000	-1.356069000
H	2.702411000	1.982098000	-2.178827000
H	3.934539000	0.899572000	-1.507860000
H	2.229967000	0.460394000	-1.409675000
C	3.963849000	3.110837000	-0.018824000
H	3.782961000	3.862762000	-0.792513000
H	4.068988000	3.612110000	0.947386000
H	4.922061000	2.633592000	-0.239060000
C	3.208624000	1.052291000	1.146728000
H	4.204620000	0.629819000	0.978354000
H	3.210893000	1.566906000	2.111785000
H	2.506789000	0.222733000	1.197023000
